# Evaluating Management of Extra-Abdominal Desmoid Fibromatosis: A Retrospective Analysis of Treatments, Outcomes and Recurrence Patterns

**DOI:** 10.3390/curroncol32060320

**Published:** 2025-05-30

**Authors:** Vidhi Saraf, Hariharan Triplicane Dwarakanathan, Al-Muaayad Al-Abri, Ioanna Nixon, Sarah Vaughan, Ashish Mahendra, Sanjay Gupta

**Affiliations:** 1 Wolfson Medical School, Glasgow University, Glasgow G12 8QQ, UK; 2830641s@student.gla.ac.uk (V.S.); 2510081a@student.gla.ac.uk (A.-M.A.-A.); 2 Glasgow Royal Infirmary, Glasgow G4 0SF, UKashish.mahendra@nhs.scot (A.M.); 3 The Beatson West of Scotland Cancer Centre, Glasgow G12 0YN, UK; ioanna.nixon@nhs.scot

**Keywords:** desmoid fibromatosis, active surveillance, surgical intervention, virtual follow up, patient-specific treatment

## Abstract

**Background:** Desmoid fibromatosis (DF) is a rare, locally aggressive soft tissue tumour with unpredictable clinical behaviour. Historically, treatment has involved surgery; however, contemporary guidelines, such as those from the Desmoid Tumour Working Group, advocate active surveillance. This article reviews current perspectives on DF, focusing on epidemiology, pathogenesis, treatment strategies, emerging research directions and cost effectiveness based on our experience at the West of Scotland Musculoskeletal Oncology Service, Glasgow Royal Infirmary (GRI). **Methodology:** We reviewed 101 patients diagnosed with desmoid fibromatosis between 2010 and 2024. A review of patient records was conducted to gather information on demographics, date of diagnosis, prior treatment, treatment initiation, intervention types, imaging intervals, follow-up duration, recurrence rate for surgery and other intervention, and discharge timelines. All data was systematically organized and analyzed to assess our outcomes. **Results:** Out of 101 patients with DF in the study, 66% were females. The most common site of primary tumour was lower extremity (39.6%) followed by near equal distribution in upper extremity and trunk. Out of the total cases, 72 (71.2%) were successfully managed with active surveillance involving serial imaging and clinical reviews in accordance with European guidelines. A total of 22 patients (21%) received treatment: 10 underwent surgery alone, 2 had surgery combined with radiotherapy, 8 received only radiotherapy, 1 was treated with hormonal therapy and 1 participated in a trial with Nirogacestat. Of the seven remaining patients, six had unplanned surgery outside followed by active surveillance at GRI. One patient was on alternative treatment modality, homeopathy. The average number of MRI scans per patient was 3.11, with many patients requiring significantly more imaging. MRI surveillance varies significantly in desmoid tumours due to their heterogeneous behaviour. Active or symptomatic tumours often require more frequent scans (every 3–6 months), while stable cases may need only imaging annually or just clinical monitoring. Recurrence was noted in eight patients, all of which were related to prior surgery. The total combined cost of imaging and appointments exceeds £6500 per patient in active surveillance. **Conclusions:** We conclude that most patients with desmoid fibromatosis in our cohort were effectively treated with active surveillance, consistent with current European guidelines. Surgical management of desmoid fibromatosis in our cohort is historic and has shown a significant recurrence risk. Our study proposes a revised follow-up protocol that significantly reduces costs without compromising on patient care. We suggest a two-year surveillance period for stable disease with patient-initiated return to reduce unnecessary clinic visits, imaging and healthcare costs.

## 1. Introduction

Desmoid-type fibromatosis (DF), also known as aggressive or deep fibromatosis is a rare low grade soft tissue neoplasm. DF is defined as “a clonal fibroblast proliferation originating in deep soft tissues with infiltrative growth and the tendency to recur locally, but with no ability to metastasize” even though it may be multifocal in the same limb or body part, according to the World Health Organization (WHO) [1]. The peak age for DF is 30–40 years with female predominance [2]. Most DFs develop sporadically (non-Familial Adenomatous Polyposis), but they are 1000-fold more common among patients with Familial Adenomatous Polyposis (FAP) [3]. Risk factors include pregnancy, prior surgery and trauma.

The main modality of imaging in DF is MRI, which can be used for diagnosis, local staging and follow-up [4,5]. A DF’s signal intensity varies with the proportion of tumour components (collagen fibres, spindle cells and extracellular matrix). MRI is indispensable in initial diagnosis and managing desmoid fibromatosis, with contrast enhancement providing valuable information on tumour activity and treatment response. However, non-contrast MRI may suffice for follow-up in stable cases and decision on contrast should be made on a case-by-case basis. Contrast (Gadolinium) in MRI helps differentiate active (hypercellular and enhancing) from fibrotic (low enhancement) components. It also aids in distinguishing desmoids from other soft tissue tumours (e.g., sarcomas, which may show more uniform enhancement). On MRI T2-weighted hyperintensity indicates active disease (cellular-rich tissue), while hypointensity suggests fibrosis/inactive disease [6,7,8]. A DF cannot be differentiated from other soft tissue tumours solely based on imaging. The differentials include lymphoma, pleomorphic sarcoma, fibrosarcoma, and giant cell tumour of the tendon sheath [9,10] A histopathologic confirmation of DF is mandatory before starting treatment [11]. An expert soft tissue pathologist should confirm the diagnosis of DF.

A mutational analysis should additionally be performed in all DF biopsy specimens to confirm diagnosis and guide further treatment. Mutations of CTNNB1 and APC are mutually exclusive in DF, hence, detecting a somatic mutation of CTNNB1 can exclude the presence of a syndromic condition and vice versa with APC mutation [12].

DF management has changed dramatically since MacFarlane of the Glasgow Royal Infirmary described them in 1832 [13]. DF’s can have unpredictable course with the possibility that tumours may regress spontaneously in 20% to 30% of patients after two to three years of follow-up [14].

A paradigm shift has occurred from surgical management to active surveillance due to cumulative evidence supporting the latter [15,16]. The local control rates for primary disease were reported as 75% for surgery, 78% for surgery combined with radiotherapy (RT), 85% for RT alone, and 78% for active surveillance [17]. For patients with recurrent disease, active surveillance demonstrated significantly better local control outcomes compared to surgery [18].

According to the most recent guidelines of the Desmoid Tumour Working Group, active surveillance is recommended for most patients with DF [11,12,19]. MRI (or CT if MRI is not possible) and clinical symptoms should be monitored for at least 2–3 years at 3-month to 6-month intervals, then every 6–12 months thereafter [12,19].

A progression of symptoms and/or persistent interval growth are indications that active management is required. When active management of DF is necessary, surgery can be considered following medical management, provided surgical morbidity is low like in abdominal wall DF. In selected cases, radiotherapy can be considered if surgery is not an option [11,12,16,19,20,21].

New treatment modalities are desperately needed when active surveillance fails and/or recurrence occurs after surgery. Quality of life should be considered when choosing any strategy. This study aimed to outline the historical and current treatment modalities for patients with desmoid fibromatosis at GRI.

## 2. Materials and Methods

In this retrospective observational study, 101 patients with desmoid fibromatosis from 2010 to 2024 were included. The patients were identified from a database of over 6000 cancer patients managed at the MSK Oncology Service at GRI during the study period. Patients with biopsy-confirmed fibromatosis but insufficient data in the database were excluded.

A review of patient records was conducted to gather information on demographics, date of diagnosis, treatment initiation, intervention types, imaging intervals, follow-up duration, time of progression for active surveillance, recurrence rate for surgery and other intervention, and discharge timelines. All data was systematically organized and analyzed to assess adherence to European guidelines.

Every case was discussed in a multidisciplinary treatment team and patient-specific treatment options were determined. Patients were treated with active surveillance, surgical resection, radiotherapy, hormonal therapy, other emerging treatments, or a combination of various treatment options. Medically managed patients were treated at the Beatson Cancer Institute.

All patients had consented in advance to their details being used for research purposes. All data was anonymized. Continuous variables (e.g., tumour size and age) are reported as mean ± standard deviation (SD) for normally distributed data and median (range) for non-parametric data. Categorical variables for recurrence were analyzed using Fisher’s Exact Test. *p* < 0.05 was considered to be significant.

The Recurrence in desmoid fibromatosis is defined by either radiological or clinical progression of the disease. Radiological recurrence includes an increase in tumour size in a previously stable or resected lesion, the development of new tumour foci at or near the original site (local recurrence), or the reappearance of disease following complete resection (R0/R1). Clinical recurrence is characterized by new or worsening symptoms, such as pain, functional impairment, or compression of adjacent structures (e.g., nerves or blood vessels), even if imaging changes are minimal. Symptomatic progression alone may justify intervention, regardless of radiological findings. Both criteria help guide decisions on whether to continue surveillance or initiate active treatment [22].

To estimate the direct costs associated with active surveillance (AS), we adopted a conservative costing approach based on current NHS tariffs, focusing on imaging and clinic visit expenses. The study assumes a 10-year follow-up period. The current cost per MRI was £130. Based on current NHS tariffs, each appointment costs approximately £233. Costs are based on current NHS pricing and are assumed to remain relatively stable over the follow-up period. Sensitivity analysis accounting for inflation or healthcare pricing changes was not included in this preliminary cost estimate but is recommended for future economic evaluations.

## 3. Results

**Figure 1:**
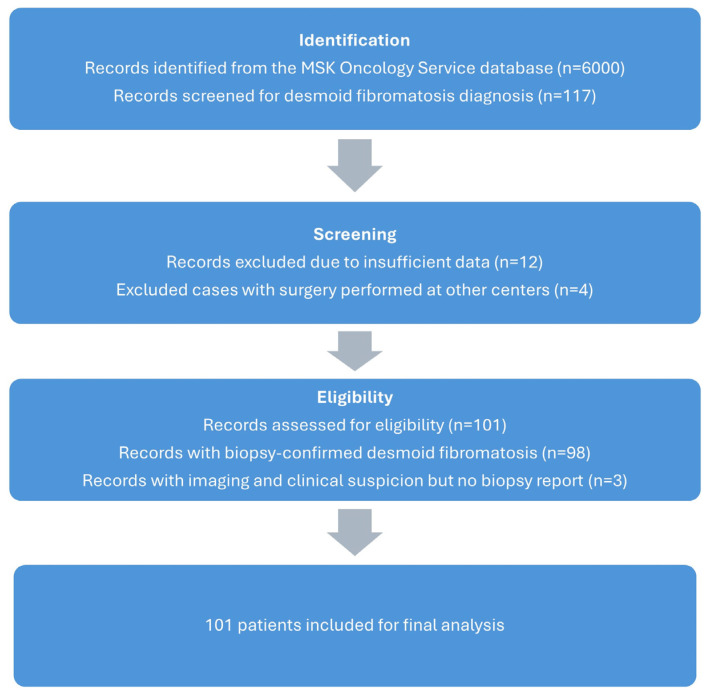
Consort flow diagram of our study group.

A total of 101 patients with desmoid fibromatosis were included in this retrospective study (Figure 1). The demographic and clinical characteristics of the patients are summarized in Table 1. The study group comprised 34 males (33.7%) and 67 females (66.3%).

The upper extremities were the site of primary tumours in 30 cases (29.7%), the lower extremities in 40 cases (39.6%), and the trunk in 31 cases (30.7%). Diagnosis was confirmed by biopsy and MRI for all patients, with an average referral-to-diagnosis time of one week. Three patients underwent biopsy, but data could not be retrieved; they have been managed as DF’s based on clinical radiological information in the database and have been included in the study. Patients with imaging and biopsy-proven diagnosis from other centres did not undergo additional imaging.

**Figure 2:**
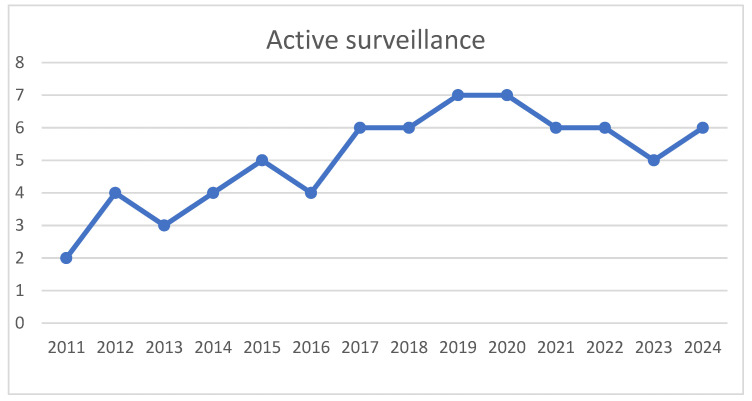
Increasing trend of active surveillance noted in study group (*x* axis–time, *y* axis–number of patients treated by active surveillance).

MRI played a critical role in both diagnosis and ongoing surveillance, with patients undergoing an average of 3.1 scans each, and a median of 3 scans (range: 0–21). However, imaging requirements varied, with some patients receiving 10 or more MRI scans due to routine imaging at each follow-up or investigations prompted by suspected disease progression. Table 2 shows the various treatment strategies used in the study group. Of the 101 patients included in the study, 72 (71.2%) were successfully managed with active surveillance (Figure 2), which entailed serial imaging and clinical reviews in accordance with Desmoid Tumour Working Group guidelines. The duration of follow-up ranged from 1 month to 13 years, with only 15 patients having a follow-up period of less than 2 years. The median follow up is of 2 years. Among those under active surveillance, 3 patients required surgical intervention due to disease progression and worsening symptoms. Notably, 39 patients (54.16%, active surveillance n = 72) demonstrated no evidence of disease progression with a minimum follow-up duration of 5 years. All these patients at our institute were followed as per European guidelines for active surveillance.

Fourteen patients (13.8%, total patients n = 101) underwent surgical intervention at the time of presentation, with 10 surgeries performed at GRI and 4 at other centres. Additionally, two patients received adjuvant radiotherapy due to positive surgical margins. Unplanned excisional biopsies were performed in two patients at external centres, and both are currently under follow-up. At our institute, the primary indications for surgery included symptomatic presentation and inconclusive pre-operative histological diagnosis.

At our centre, the most common reasons for surgery historically were pain, and pressure-related symptoms, noted in nine patients. In one case, surgery was performed due to diagnostic uncertainty between desmoid fibromatosis and low-grade fibromyxoid sarcoma based on preoperative histology.

Additional interventions included radiotherapy as a standalone treatment in eight patients (7.9%), hormonal therapy in one patient (1.0%) and enrolment in a clinical trial for Nirogacestat in one patient (1.0%). Recurrence was observed in 8 of the 22 patients who received treatment (Table 3 and Table 4). Six patients who had undergone unplanned surgery at other centres were subsequently managed at GRI with surveillance. One patient chose to have homeopathic treatment. Surgery was found to be a significant risk factor (*p* < 0.0002) for recurrence as all the recurrence patients had prior surgery.

The five patients managed with watch-and-wait (active surveillance) in our recurrent subgroup represent practice shifts, consistent with contemporary evidence demonstrating the safety of observation for stable/asymptomatic disease. We acknowledge that current standards would prioritize non-operative strategies—such as systemic therapy or radiotherapy—for most recurrences, particularly given the morbidity associated with repeated resections (e.g., amputation in one case).

In our cohort, two patients underwent amputations—one performed at our centre in 2023 (above-knee amputation for recurrent fibromatosis in a previously irradiated left lower leg, complicated by prior lymphoma history), and another referred postamputation from another NHS trust for ongoing surveillance. Notably, these amputations resulted from overly aggressive surgical attempts to achieve negative margins, leading to significant morbidity without clear oncologic benefit. This highlights the importance of structure-preserving approaches as the primary goal in desmoid management, given the tumour’s non-metastatic nature and the feasibility of long-term control with less radical strategies (e.g., systemic therapy or watchful waiting). Avoiding unnecessary mutilating surgery is critical to preserving function and quality of life.

## 4. Discussion

Desmoid fibromatosis (DF) presents a challenging clinical entity characterized by its unpredictable natural history and variable response to treatment. In this analysis of 101 patients over a 14-year period, we evaluated the evolution of treatment strategies, clinical outcomes, and recurrence patterns at our institution. Our findings align with the recommendations of the Desmoid Tumour Working Group guidelines, while also highlighting novel research questions that could be more effectively addressed through meta-analyses or randomized controlled trials (RCTs).

Historically, surgery was the primary treatment for desmoid fibromatosis (DF), reflecting earlier practices prior to the adoption of active surveillance as the standard first-line management. Our cohort illustrates this trend, with a significant reduction in surgical interventions after the publication of the European guidelines in 2020. These guidelines, based on a 2017 consensus meeting, recommend an “active surveillance” or “watch-and-wait” approach as the preferred initial management strategy [11,12,19,23]. This shift is evident in our data, as only two patients underwent surgery after 2020.

Radiotherapy was utilized in eight patients, with an additional two receiving combined surgery and radiotherapy. This approach was particularly effective for older patients, those with comorbidities, or individuals with rapidly progressing tumours. Notably, recurrence rates following radiotherapy alone were comparable to those seen with combined surgery and radiotherapy, underscoring the importance of minimizing morbidity by avoiding overly aggressive surgical interventions. In our cohort, two patients underwent amputations, one of which was necessitated by recurrence within a previously irradiated field. These findings highlight the critical need for structure-preserving procedures in the management of DF to reduce treatment-associated morbidity.

There is a higher prevalence of desmoid fibromatosis (DF) in females in the existing literature, which suggests that hormonal influences, particularly estrogen, play a significant role. Additionally, antecedent trauma and pregnancy have been implicated as contributing factors, particularly in the development of anterior abdominal wall desmoids in women of childbearing age [24]. These observations reinforce the importance of considering hormonal and demographic factors in the etiopathogenesis and management of DF.

MRI is the primary imaging modality for DF evaluation in our cohort, offering detailed soft tissue characterization. However, its limitations in distinguishing DF from soft tissue sarcomas (STS) based solely on imaging features mean tissue biopsy remains the gold standard for definitive diagnosis. Immunohistochemical staining for β-catenin and CTNNB1 mutation analysis are crucial for distinguishing DF from other soft tissue tumours [25]. Hence the diagnosis requires a trained musculoskeletal radiologist and pathologist to be part of MDT and help to formulate a patient specific treatment plan.

The average number of MRI scans per patient was 3.11, with many patients requiring significantly more imaging either as part of active surveillance, disease progression or to guide interventions. Our centre previously followed the recommendation for intensive imaging during the initial surveillance period, with monthly scans for the first two months, followed by imaging every three months during the first year, biannual scans until the fifth year and annual imaging thereafter [11,12].

We propose a streamlined follow-up protocol designed to enhance patient care and efficiency while substantially reducing healthcare costs. Rather than adhering to the conventional 10-year surveillance framework, we recommend a focused 2-year monitoring period comprising an initial consultation at baseline (day 0), followed by MRI evaluations at 6, 12 and 24 months. In the absence of significant clinical or radiological concerns, patients would subsequently transition to a patient-initiated follow-up model, thereby discontinuing routine imaging and eliminating unnecessary scheduled visits. This approach aligns with observations that disease progression is most common within the first three years, with stabilization typically occurring between 14 and 19 months after diagnosis.

Assuming a 10-year follow-up period in line with European guidelines, each patient undergoes an average of 18 MRI scans, with the following distribution: five scans in year one, eight scans from years two to five and five scans from years six to ten. At a cost of roughly £130 per MRI scan, this translates to a total imaging cost of £2340 per patient. Assuming an equal number of clinic visits throughout the follow-up period, with each consultant-led clinic appointment costing £233 as per current NHS Scotland figures, the total combined cost of imaging and appointments exceeds £6500 per patient.

Under our proposed guidelines, with follow-up limited to two years and patient-initiated returns thereafter, each patient undergoes four MRI scans and four consultant visits. The total cost for imaging and consultations is £1452 per patient, resulting in a savings of £5048 per patient compared to the standard 10-year European follow-up model. In a publicly funded healthcare system such as the NHS, this represents a significant allocation of resources.

Our study hints that routine long-term follow-up may be unnecessary for asymptomatic patients, provided they have access to specialist care if symptoms recur, as there was no demonstrable change in management plan. Reducing the frequency of follow-up visits and imaging in carefully selected stable cases could significantly lower healthcare costs without compromising quality of care. For select patients, tailoring follow-up protocols to prioritize resource utilization offers a practical approach to optimizing outcomes while conserving healthcare resources.

We propose leveraging the successful frameworks of the Virtual Fracture Clinic (VFC) model, developed at the Glasgow Royal Infirmary (GRI), and the virtual musculoskeletal (MSK) radiology multidisciplinary team (MDT) used for sarcoma referral triage, to develop a virtual clinic model for active surveillance of desmoid fibromatosis (DF) patients. These models demonstrate the potential of remote, patient-initiated follow-ups to reduce costs and improve healthcare efficiency without compromising clinical outcomes [26,27].

At the Beatson Cancer Centre, we employ a stepwise, individualized approach to desmoid fibromatosis, guided by current national recommendations (Sarcoma Specialist Network). While most patients are managed with active surveillance at presentation, we discuss all therapeutic options during initial counselling. For selected cases requiring intervention, we begin with well-tolerated first-line hormonal therapy (tamoxifen 20 mg twice daily, often combined with NSAIDs like sulindac or celecoxib) for disease stabilization, accompanied by regular clinical and radiological monitoring. For cases demonstrating rapid progression, or hormonal therapy failure, we escalate to systemic treatments.

Chemotherapy options include weekly IV methotrexate/vinblastine (1–2 years duration, offering ~50% Response Evaluation Criteria in Solid Tumours (RECIST) response and ~80% symptomatic improvement, with median PFS ~6 years), oral vinorelbine (less commonly used), or full-dose doxorubicin-based regimens as a last resort for refractory or life-threatening disease. Tyrosine kinase inhibitors (TKIs), particularly sorafenib, which has shown excellent responses in our experience, or imatinib (~50% disease control at two years)—are preferred for chemo-refractory or selected cases. Our philosophy emphasizes avoiding overtreatment, as many desmoids stabilize spontaneously; active surveillance is a valid strategy for indolent disease. Medical therapy balances disease control with quality of life, reserving aggressive therapies for truly progressive or high-risk scenarios.

Our study identified surgery alone as a significant risk factor for recurrence. While surgical excision remains an option for very select cases, the emphasis has shifted towards less invasive modalities. One patient in our cohort was considered for Nirogacestat, a promising gamma-secretase inhibitor, while two patients recently at our institute underwent cryotherapy, reflecting the emerging role of novel therapeutic approaches in managing DF. This approach is particularly advantageous due to its low complication rates and ability to provide durable tumour control and symptom relief in the short to medium term.

Adhering to the full five-year follow-up protocol may be unnecessary for patients who are symptom-free and whose fibromatosis is stable, or which show signs of regression. These patients could typically re-engage with clinical services only if symptoms such as inadequate pain control or tumour progression occur.

This analysis has limitations that should be considered when interpreting the results. First, treatment patterns evolved over time in keeping with changing international guidelines, making direct comparisons between historical and contemporary approaches challenging. Second, as a tertiary referral centre, our cohort includes patients who were referred from other NHS trusts after initial management elsewhere, as well as those lost to follow-up when returning to their local health boards. This mobility of patients (being “in and out of area”) may have led to incomplete long-term outcome data for some cases. Additionally, our database has gaps due to loss to follow-up, particularly for patients managed conservatively who may have sought care elsewhere as their condition stabilized. These factors could introduce selection bias and affect the generalizability of our findings regarding treatment efficacy and progression rates. Future prospective studies with standardized follow-up protocols would help address these limitations.

Larger, adequately powered studies are needed to evaluate whether it may be feasible to discharge patients after an initial diagnosis and the implementation of a virtual “watch and wait” strategy, provided they have direct access to specialist services in case of symptom recurrence. Such an approach could reduce unnecessary clinical visits, imaging, and associated healthcare costs without compromising patient care. Further research is essential to assess the safety and efficacy of this potential follow-up model.

## 5. Conclusions

Our study demonstrates that most desmoid fibromatosis patients were successfully managed with active surveillance, consistent with current European guidelines. No disease progression was observed in the surveillance cohort, leading to the adoption of a two-year follow-up protocol, after which patients are discharged with the option for patient-initiated follow-up. This individualized, quality-of-life–focused strategy minimizes treatment morbidity and clinic burden and is now our recommended approach. Further research is needed to assess the safety and cost-effectiveness of virtual follow-up models.

## Figures and Tables

**Table 1:** Patient demographic and clinical profile data.

Variables	Number n (%)
Gender	
Female	67 (66.3)
Male	34 (33.7)
Site	
Lower extremity	40 (39.6)
Upper extremity	30 (29.7)
Torso	31 (30.7)
Symptoms at presentation	
Swelling	99 (98.01)
Pain	2 (1.9)
MRI	101
Pre-treatment biopsy performed	101
Biopsy report available	98

**Table 2:** Treatment strategies at presentation in the study group.

Treatment Strategies at Presentation	Number n = 101 (%)
Active surveillance	72 (71.2)
Surgery at GRI	10 (9.9)
Surgery external surveillance at GRI	4 (3.9)
Surgery and radiotherapy	2 (1.9)
Radiotherapy	8 (7.9)
Unplanned excision biopsy external, surveillance at GRI	2 (1.9)
Homeopathy	1 (1)
Hormonal therapy (Tamoxifen)	1 (1)
Nirogacestat (clinical trial)	1 (1)

**Table 3:** Risk factors for recurrence in study group, RT: radiotherapy.

Recurrence	Yes	No	*p*-Value
Gender			0.58
Female	6	61	
Male	2	32	
Site			0.65
Torso	2	29	
UL	4	26	
LL	2	38	
Treatment			0.0002
Surgery	8	6	
Non-surgical treatment	0	14	
Initial margin positive excision	2	0	0.49

**Table 4:** Details of patients with recurrence.

Patient	Initial Surgery	Recurrence Timeline	Management of Recurrence	Outcome/Follow-Up
1	2009	2011	Radiotherapy (RT)	No recurrence after RT. PFS till 2024
2	2010	2012	Tamoxifen + NSAIDs	Stable on tamoxifen
3	2015	2017	Active surveillance	Stable disease
4	Unplanned resection elsewhere—2016	2017	Active surveillance	Stable disease
5	2010—operated elsewhere	2024	Active surveillance	Stable disease
6	Multiple surgeries (2002, 2006 and 2008)	2008	Active surveillance	Stable disease
7	2011	2015	Surgery + RT	No recurrence
8	2014	2015	Active surveillance	Stable disease

## Data Availability

The original contributions presented in this study are included in the article. Further inquiries can be directed to the corresponding authors.
